# Early antagonism of cerebral high mobility group box-1 protein is benefit for sepsis induced brain injury

**DOI:** 10.18632/oncotarget.21502

**Published:** 2017-10-05

**Authors:** Chao Ren, Ya-Lin Tong, Jun-Cong Li, Ning Dong, Ji-Wei Hao, Qing-Hong Zhang, Yong-Ming Yao

**Affiliations:** ^1^ School of Medicine, Nankai University, Tianjin 300071, People's Republic of China; ^2^ Trauma Research Center, First Hospital Affiliated to The Chinese PLA General Hospital, Beijing 100048, People's Republic of China; ^3^ Department of Burns and Plastic Surgery, The 181st Hospital of Chinese PLA, Guilin 541002, People's Republic of China; ^4^ State Key Laboratory of Kidney Disease, The Chinese PLA General Hospital, Beijing 100853, People's Republic of China

**Keywords:** HMGB1, sepsis, lateral ventricles, antagonists, brain injuries

## Abstract

Sepsis induced brain injury acts as an acute complication and accounts for deterioration and high mortality rate of septic condition. HMGB1 is a late inflammatory mediator that plays a critical role in brain dysfunction and diseases. However, the role of HMGB1 in sepsis induced brain dysfunction remains intricate. The current study investigated the effect of HMGB1 on brain injury in septic mice model with intracerebroventricular injection of BoxA (a specific antagonist of HMGB1). The expression of HMGB1, morphological changes of brain tissues, apoptosis of brain cells, and alteration of behavior were determined. The expressions of HMGB1 in cortex, hippocampus, and striatum were significantly enhanced in the sepsis group when compared with the sham group. In septic conditions, brain tissues showed significant abnormalities in tissue structure, and increased apoptosis of brain cells which was caspase-3 dependent. Septic mice showed suppression of locomotor activity and impairment of memory and learning. Neutralizing brain HMGB1 significantly improved brain injury and apoptosis of brain cells, and further ameliorated disturbed locomotor activities and damaged memory and learning. However, no significant improvement of survival rate was seen after inhibiting central HMGB1. These results reveal that HMGB1 is a potential target for ameliorating sepsis induced brain injury with early antagonizing.

## INTRODUCTION

Sepsis is one of the leading causes of mortality in intensive care units, manifested by uncontrolled inflammation and multiple organs failure. Organ dysfunction, including heart, liver, lungs, kidneys and brain, accounts for high mortality rate and limited therapeutic measures after the onset of sepsis [1–5]. Brain injury occurs in the early phase of sepsis, and contributes to the progression of sepsis [6]. Patients who suffer from sepsis associated brain dysfunction might experience acute changes of feeling and mental status, including sleep deprivation, restlessness, inattention, and different levels of conscious disorders that range from delirium to coma [7]. In animal models of sepsis, mice also show disrupted rhythms of temperature and activity, loss of body weight, and reduced social exploration, which are not recovered after survived from serious stage [8]. Further researches have documented that brain might be the first organ affected by systemic inflammation [9]. However, little progress is achieved in exploring prompt diagnosis and effective interventions partly owing to indistinct mechanisms of pathogenesis and development of brain injury compromised by sepsis settings.

Neuro-inflammation is one of the key factors for the development of brain injury in septic condition [10]. The apoptotic neurons were mainly affected by neuro-inflammation, as documented by Pfister and colleagues [10]. Therefore, neuro-inflammation is definite a great threat to brain cells, which is characterized with increased infiltration of inflammatory cells, excessive production of pro-inflammatory cytokines, such as interleukin-1β (IL-1β), tumor necrotic factor-α (TNF-α), IL-6, and high mobility group box-1 protein (HMGB1), and over-activation of microglial cells [3, 11]. Pro-inflammatory mediators are key components of neuro-inflammation, and act as potential therapeutic targets for brain injury. TNF-α, for instance, is documented to affect cerebral edema and neuron apoptosis in septic response, which is vanished after TNFR1 deficient [12].

HMGB1 is identified as a later pro-inflammatory mediator after released by necrotic cells and secreted by immune cells, and plays a critical role in brain dysfunction and diseases [13]. In animal models of ischemic brain injury, for example, HMGB1 expression shows significantly enhancement from 1 to 7 days post surgery, and is associated with increased production of inflammatory cytokines and alteration of neurological function [14]. Early translocation and release of HMGB1 are noted in experimental subarachnoid hemorrhage, which initiate at 2 hours and peak on day 1 after injury [15]. Inhibiting the expression and the action of HMGB1 significantly attenuate neuro-inflammation, and further ameliorate brain injury [14, 16]. In septic animal models, inhibition of serum HMGB1 shows great benefits for ameliorating brain injury, as evidenced by improving learning and memory, and alleviating damaged hippocampal structure [17]. However, the expression and the potential role of HMGB1 in different brain regions and during early phase of sepsis have not been elucidated yet.

## RESULTS

### HMGB1 expressions in various brain regions were significantly enhanced in sepsis

**Figure 1:**
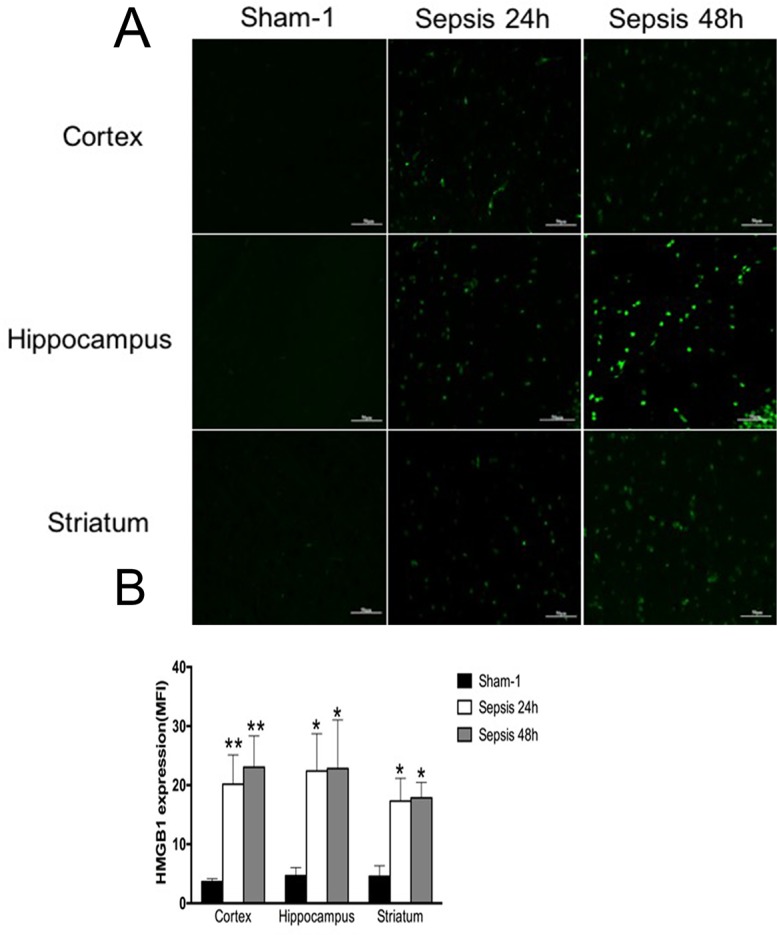
Expressions of HMGB1 in various brain regions measured by immunofluorescence staining **(A)** Brain tissues of cortex, hippocampus, and striatum showed significantly enhanced expression of HMGB1 in the sepsis group compared with the sham group, as presented with green fluorescent (n=6). **(B)** Statistical results for immunofluorescence staining of HMGB1 expression (vs. the sham group: ^*^ *P*<0.05, ^**^ *P*<0.01).

**Figure 2:**
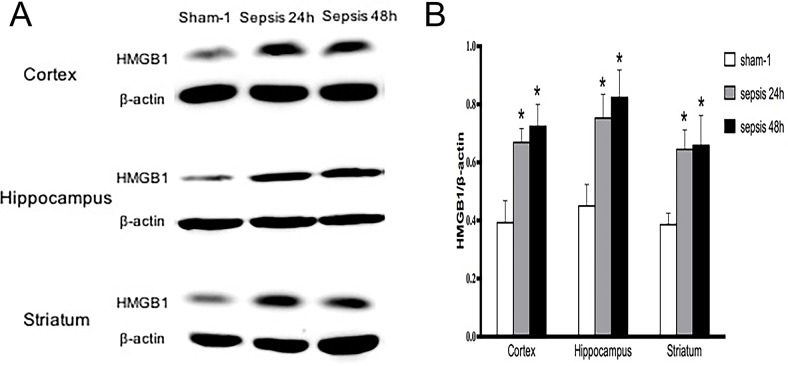
Expressions of HMGB1 in various brain regions determined by Western blotting **(A)** HMGB1 protein expressions of cortex, hippocampus and striatum showed markedly high levels in the sepsis group when compared with these in the sham group (n=6). **(B)** Statistical results of HMGB1 protein expressions by Western blot (vs. the sham group: ^*^ *P*<0.05).

In comparison with the sham group, HMGB1 protein levels of various brain regions were all significantly elevated in the sepsis group, as shown by immunofluorescence results (Figure 1A, 1B). It was also shown evidence of enhanced HMGB1 expression by Western blotting in the setting of sepsis (Figure 2A, 2B).

### Central blockade of HMGB1 with BoxA ameliorated sepsis-induced pathological changes of various brain tissues

**Figure 3:**
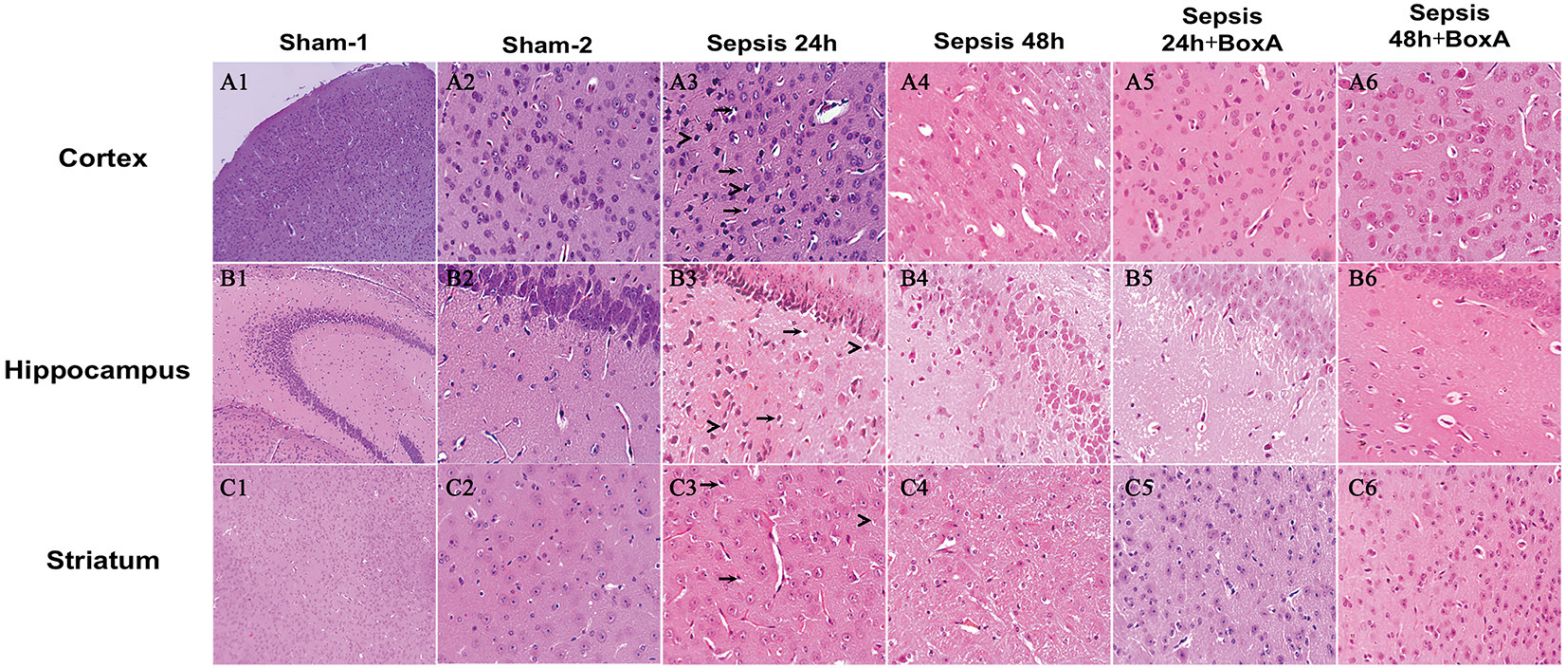
HE staining of brain tissues for assessing morphological changes (magnification: A1, B1, C1: ×100, A2-6, B2-6, C2-6: ×400) Pathological sections revealed disorganized structure, edema of brain cells, and increased infiltration of lymphocytes in the sepsis group. Antagonizing HMGB1 with 1 μg BoxA obviously attenuated the pathological alterations, including clear structure of brain tissues, reduced edema of brain cells, and decreased infiltration of lymphocytes in comparison to the sepsis group.“>” for labeling edema cells, “→” for labeling lymphocytes, sham 1: sham CLP group, sham 2: sham ICV injection group, sepsis 24h: 24h after CLP, sepsis 48h: 48h after CLP.

As shown by HE staining (Figure 3: A1, A3, A4, B1, B3, B4, C1, C3, C4), remarkable damage of the cortex, hippocampus, and striatum was observed in the sepsis group as compared to the sham group, including disorganized structure, edematous brain cells, and increased infiltration of lymphocytes. However, brain tissues in the sepsis group treated with BoxA revealed improvement of pathological changes, manifested by well-organized brain structure, reduced brain edema, and decreased lymphocytes infiltration in comparison to the sepsis group (Figure 3: A5-6, B5-6, C5-6).

### Sepsis induced increases in S100β and neuron-specific enolase (NSE), and were diminished by antagonizing brain HMGB1

**Figure 4:**
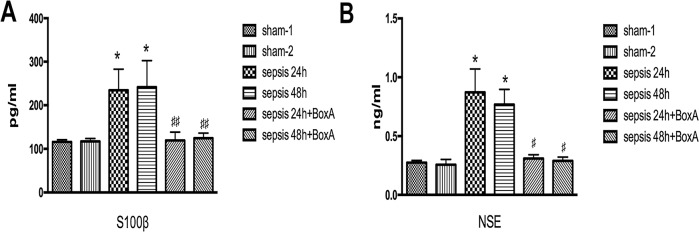
Effects of antagonizing cerebral HMGB1 on serum levels of S100β and NSE **(A)** Serum S100β level was significantly elevated in the sepsis group compared with the sham group, while sepsis with BoxA injection group showed lower level of S100β than that in the sepsis group at the same time point (n=6). **(B)** In comparison to the sham group, serum NSE level showed a significant increase in the sepsis group. However, intracerebroventricular injection of BoxA reduced the NSE level by inhibiting HMGB1 as compared with the sepsis group (n=6, vs. the sham-1 group: ^*^ *P*<0.05, ^**^ *P*<0.01; vs. the sepsis group: ^#^*P*<0.05, ^##^*P*<0.01).

Serum S100β and NSE were common markers for brain injury in septic response [18, 19]. ELISA results exhibited higher levels of serum S100β and NSE in the sepsis group than those of the sham group (n=6, *P*<0.05, Figure 4). In the sepsis with BoxA treatment group, however, S100β and the NSE levels were both decreased in comparison to the sepsis group (n=6, *P*<0.05).

### Antagonizing HMGB1 reduced apoptosis of the brain cells that were compromised by CLP, accompanied by alteration of caspase-3 activity

**Figure 5:**
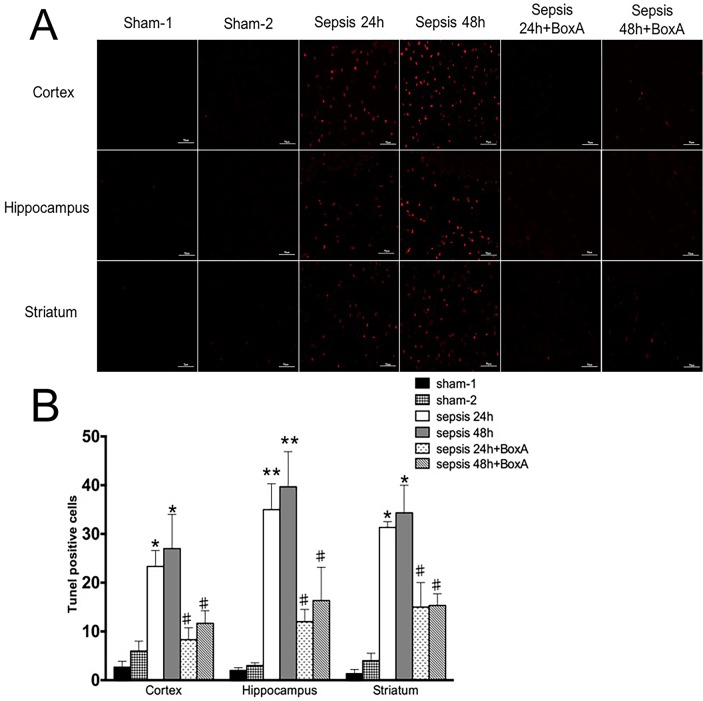
TUNEL staining for assessment of apoptosis of brain cells **(A)** Apoptosis of brain cells was significantly enhanced in the sepsis group compared with the sham group, as shown by red points (n=6). Antagonizing brain HMGB1 reduced the number of apoptotic cells of various brain regions in comparison to those in the sepsis group(n=6). **(B)** Histogram showed clearly difference of TUNEL positive cells in various groups (vs. the sham-1 group: ^*^ *P*<0.05, ^**^ *P*<0.01; vs. the sepsis group: ^#^*P*<0.05).

With TUNEL staining, we found that exposed mice showed markedly increase of apoptosis in various brain regions when compared with those in the sham groups (Figure 5). The apoptosis was diminished when HMGB1 was inhibited, as evidenced by less positive cells of TUNEL staining than the sepsis groups.

**Figure 6:**
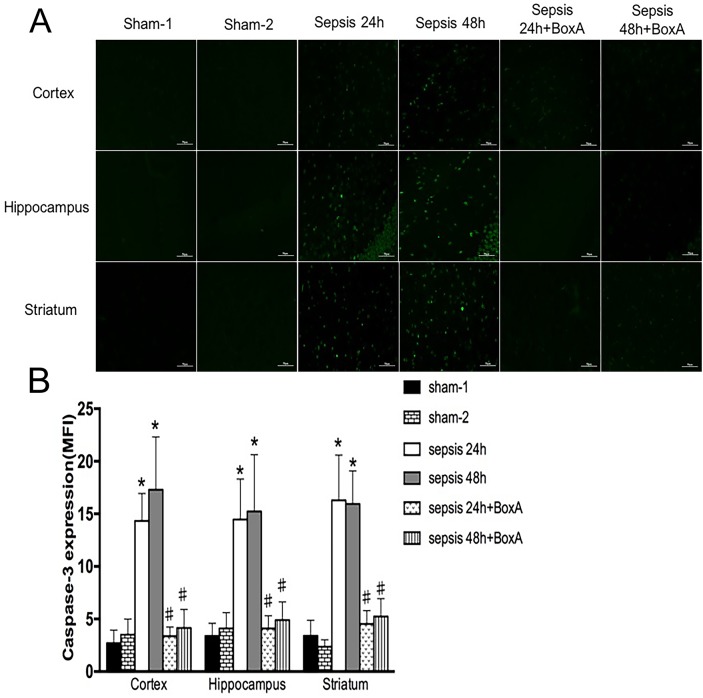
Expressions of caspase-3 in various brain regions measured by immunofluorescence staining (green) **(A)** In the sepsis group, expressions of caspase-3 in different brain regions were elevated in comparison to those in the sham group (n=6). Blockade of brain HMGB1 markedly lowered caspase-3 expressions when compared to those in the sepsis group (n=6). **(B)** Statistical results clearly showed the difference among these groups (vs. the sham-1 group: ^*^ *P*<0.05; vs. the sepsis group:^#^*P*<0.05).

Previous studies reported an important role of caspase-3 on apoptosis of brain cells [20]. In comparison with the sham group, expressions of caspase-3 in different regions were significantly enhanced in the sepsis group (Figure 6). However, antagonism of central HMGB1 reversed the elevated expression of caspase-3 following septic challenge.

### Administration of BoxA improved locomotor activity in septic mice

**Figure 7:**
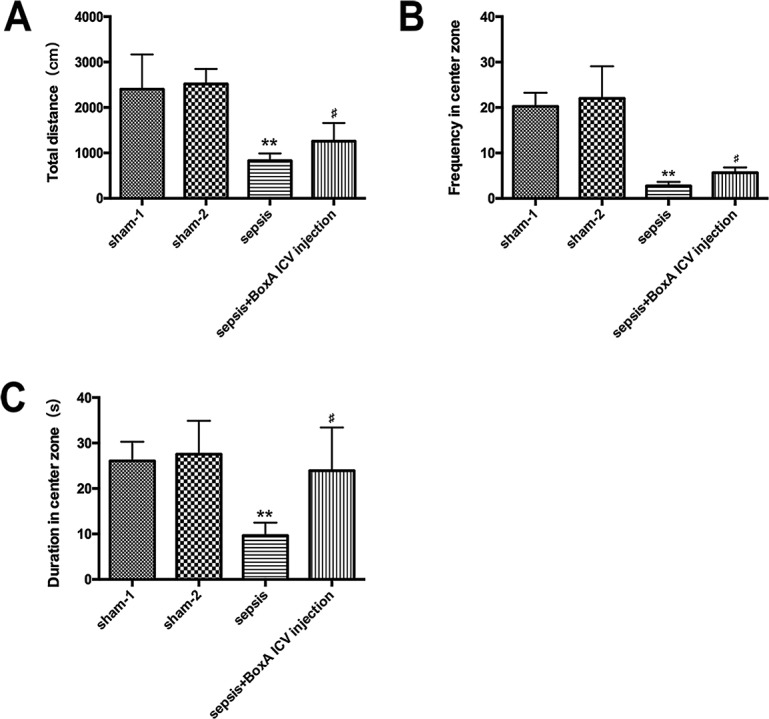
The open field test for measuring locomotor activity of mice in various groups **(A)** Sepsis resulted in a significant decrease of total distance of mice in the open filed, while this effect was abated by antagonizing brain HMGB1(n=6). **(B)** Neutralizing cerebral HMGB1 obviously improved frequency in center zone of mice that greatly suppressed in the setting of sepsis (n=6). **(C)** The duration in center zone was presented with lower level in the sepsis group than that in the sham group, while improved by injecting BoxA into right lateral ventricle (n=6, vs. the sham-1 group: ^*^ *P*<0.05, ^**^ *P*<0.01; vs. the sepsis group: ^#^*P*<0.05).

The total distances in the sepsis group revealed lower levels than those in the sham group (n=6, *P*<0.05, Figure 7A), and the frequency and duration of entry into center zone were also impaired in septic condition (Figure 7B, 7C). Antagonism of central HMGB1, however, significantly ameliorated disturbed locomotor activities (n=6, *P*<0.05).

### Anti-HMGB1 treatment ameliorated memory damage of mice in sepsis

**Figure 8:**
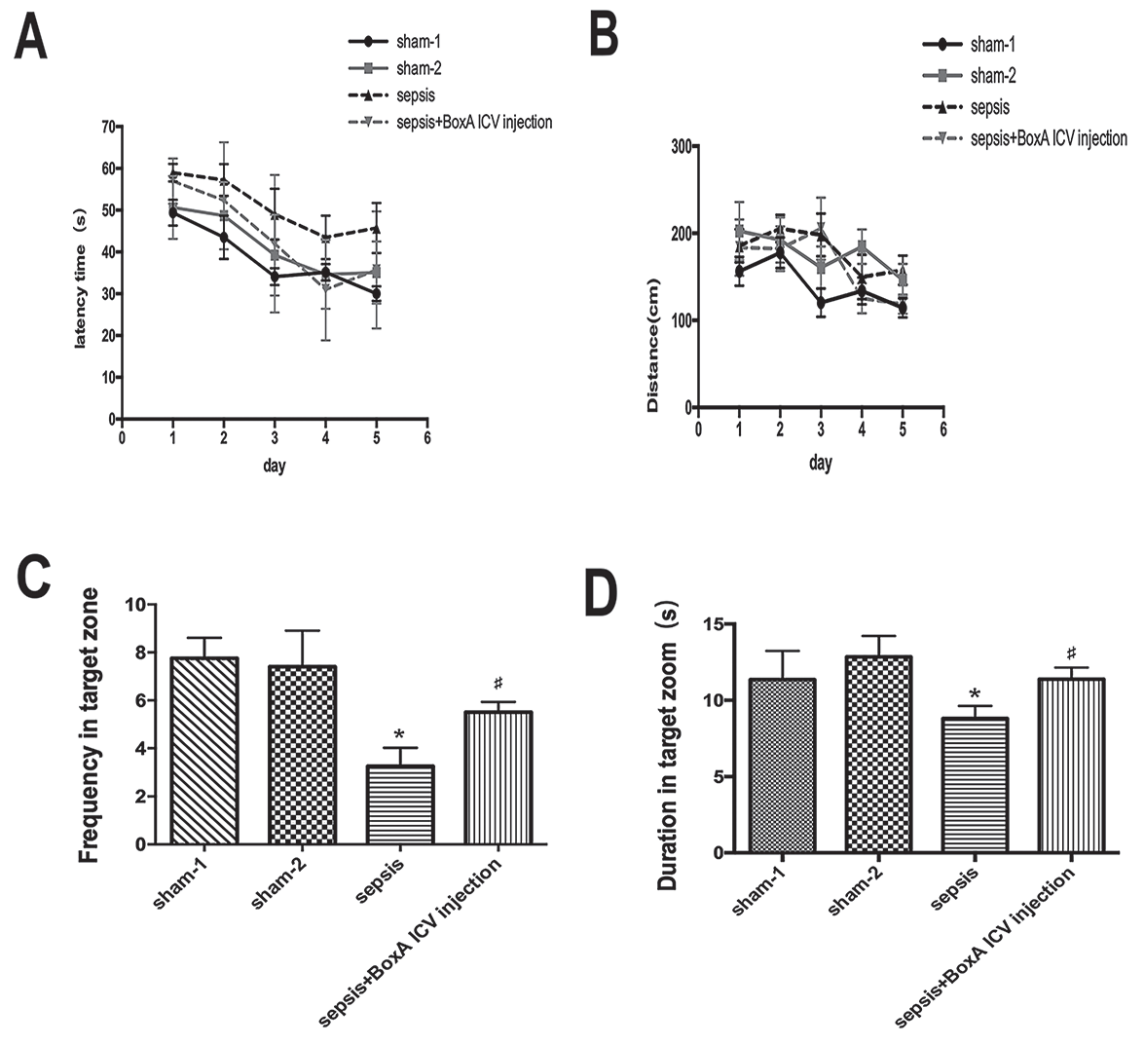
The impairment of memory and learning of mice determined by Morris water maze **(A)** The latency time to escape was increased on days 3, 4 and 5 after septic challenge. Antagonizing brain HMGB1 abated the prolonged latency time of septic mice (n=6). **(B)** Blocking cerebral HMGB1 showed no significant difference in escaped distance among the four groups (n=6). **(C)** Sepsis induced a significant decrease of frequency in target zone compared with the sham group, while these effects were vanished by intracerebroventricular BoxA injecting (n=6). **(D)** Neutralizing brain HMGB1 also improved suppressed duration in target zone of septic mice (n=6, vs. the sham-1 group: ^*^ *P*<0.05, ^**^ *P*<0.01; vs. the sepsis group: ^#^*P*<0.05).

Mice in the sepsis group exhibited increased latency time on the fifth day of training (n=6, *P*<0.05). Exposed mice were suffered from decreased frequency and duration time in target zone compared with mice in the sham group, but the abnormalities of memory and learning were markedly attenuated by neutralizing brain HMGB1 (n=6, *P*<0.05, Figure 8).

### Survival rate

**Figure 9:**
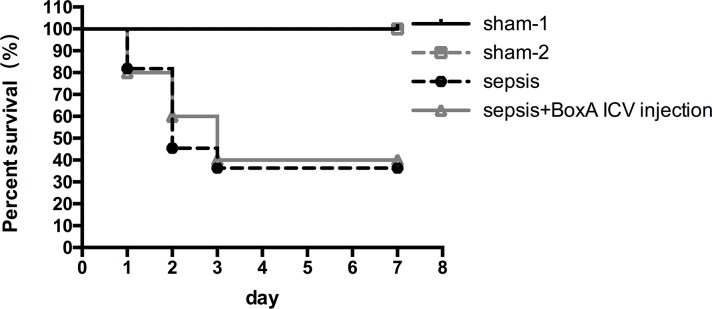
Survival rates of different groups within 7 days after CLP operations Survival rates were recorded within 7 days after CLP operation, and it was 30% in the sepsis group on 7 days post surgery(n=20). Inhibiting brain HMGB1 expression showed no remarkable difference in comparison to that in the sepsis group (n=20, *P*>0.05).

No mice died in the sham-1 and sham-2 groups. The survival rate was 30% in the sepsis group on 7 days post surgery (n=20). Nevertheless, no remarkable increase in the survival rate was found in the sepsis plus BoxA group (40%) when compared to that in the sepsis group (n=20, *P*>0.05, Figure 9).

## DISCUSSION

HMGB1 acts as an important damage-associated molecular pattern to respond injury, and shows intensive expression after initiation of sepsis [21]. In the current study, we found a marked increase of HMGB1 expression in various brain regions involving cortex, hippocampus, and striatum after septic challenge. These areas are important for maintaining homeostasis of various body functions. As reported previously, cortex and hippocampus were essential for feeling, learning and memory, but vulnerable to septic stimuli [22]. The striatum that played a significant role on motor activity was also damaged when exposed to sepsis [23]. Additionally, time points of 24 and 48 hours were chosen to observe as mice developed evident signs and notable mortality after CLP operation [24]. The marked release of brain HMGB1 might arise from various reasons: the first roots in passive release from necrotic neuronal cell that was a repository for intracellular HMGB1 [25]. Secretion from activated microglial is another source of HMGB1, which might be released quickly by cytoplasmic vesicles transportation [26]. The activation of microglial relies on inflammatory stimuli from both neural and humoral mechanisms. In addition, HMGB1 is a small molecular protein with 30 kDa that can pass through the blood brain barrier (BBB), which might contribute to the promotion of brain HMGB1 with its high level in circulation in septic patients [27].

Additionally, this study demonstrated that tissues from mice cortex, hippocampus, and striatum developed pathological damage during the process of sepsis, which presented a diffuse cerebral injury under sepsis condition. These findings could help to explain acute changes of mental status in septic mice [8]. Meanwhile, histopathological alterations were identical with those in inflammatory brain injury, as reported previously [28]. In our study, histopathological changes were improved by neutralizing brain HMGB1 with BoxA solution. The BoxA is a conserved DNA-binding domain of HMGB1 with anti-inflammatory effect. It acts as one of specific antagonists of HMGB1, and plays a protective role in sepsis by attenuating excessive inflammation. As far as we know, previous studies mainly focused on down-regulating the release and the action of peripheral HMGB1, but without any reports on antagonizing central HMGB1 directly in sepsis settings. Additionally, serum S100β and NSE both are useful markers by reflecting injury of both glial cells and neurons [19]. Nonetheless, Macedo and colleagues documented that there was lack association between serum levels of S100β and NSE and mortality in critical ill patients [29]. From the present study, antagonism of brain HMGB1 was effective in decreasing serum S100β and NSE levels, which implicating a potential therapeutic role of targeting HMGB1 in ameliorating sepsis induced brain dysfunction.

Enhanced formation of pro-inflammatory cytokines has been reported accounting for extensive apoptosis in brain tissues [3]. From our results, antagonism of cerebral HMGB1 revealed great benefits for alleviating apoptosis of brain cells, which presented the similar effects with ethyl pyruvate that was a potential inhibitor of HMGB1 [16]. However, little study illustrates the specific mechanisms of HMGB1 on apoptosis of brain cells in septic response. In the current experiments, the caspase-3 activity was obviously up-regulated under CLP operation, but markedly reversed by blocking brain HMGB1, supporting the concept that the apoptosis of brain cells compromised by HMGB1 seemed to be caspase-3 dependent.

Behavior changes are also strong evidences for determining sepsis induced brain dysfunction. Sick behavior, decreased consumption of food and water as an example, lasts for 2-3 days after sepsis initiation, while dysfunction of cognition and motor activity that acutely develops after CLP has been reported to change for 23 days or even longer [8, 17]. HMGB1 has been documented involving in suppressed cognition and locomotor activity in septic survivors [17]. However, the measures of inhibiting HMGB1 are often applied at recovery stage of septic complications. As shown in our observations, the pathological changes of brain injury developed early after onset of sepsis. Thus, we neutralized cerebral HMGB1 right after CLP surgery, and found improvements on activities and memory of mice undergone sepsis. These findings might imply a protective effect of HMGB1 antagonist on sepsis induced brain dysfunction by early administration. Serum HMGB1 concentration was markedly elevated from 8 to 36 hours, and accounted for high mortality rate of severe sepsis [30]. Targeting serum HMGB1 could significantly improve survival rate of septic mice and expand therapeutic window. Nevertheless, antagonizing brain HMGB1 didn't significantly reverse the high mortality rate induced by severe sepsis in our study. Multiple factors have been proposed to explain the discrepancy. While there is obvious difference in the concentration of HMGB1 between serum and cerebrospinal fluid, and the action of brain HMGB1 could be inhibited with low dose of BoxA which might be unable to work in the systemic circulation. Another possible explanation is that multiple organs dysfunction appears simultaneously or sequentially after the onset of sepsis, and contributes to refractory mortality. We therefore speculated that no significant difference of survival rates might turn up when septic mice only had brain injury ameliorated.

Our findings provide direct evidence of HMGB1 expression in brain tissues, and show its potential role in sepsis induced brain injury. Therefore, HMGB1 might be a promising target for preventing the occurrence and the progression of brain dysfunction, which can be addressed right after the onset of sepsis through intracerebroventricular administration of antagonists. However, the significance of brain HMGB1 expression and its mechanism concerning tissue injury remain further elucidated. Additionally, the effect of HMGB1 in neuro-inflammation should also be clarified. With regard to behavior changes, we mainly focused on changes in locomotor activity and memory. Nonetheless, alterations of emotion and social behavior are of importance to assess the severity of brain dysfunction. We are expecting to find effective therapeutic targets to improve acute brain damage by exploring the precise mechanisms underlying HMGB1 on neuro-immune interaction following septic challenge.

## MATERIALS AND METHODS

### Animals

Wild type C57BL/6 male mice (6-8 weeks old, weighing 20-22 g) were purchased from Laboratory Animal Science of Chinese Academy of Medical Sciences, Beijing. These mice were housed in standard cages with controlled temperature and humidity, 12 hours light and 12 hours dark cycle, and free access to forage and water. All experiments were conducted in accordance with the National Institutes of Health (NIH) Guidelines, and approved by the Scientific Investigation Board of the Chinese PLA General Hospital (No. SYXK2012-0014), Beijing, China.

### Cannulation of lateral ventricles

Mice were placed on the motorized stereotactic apparatus (Stoelting Co., Wood Dale, IL) under anesthetized by inhaling isoflurane (3% induction, 1.5% maintenance). The skin of mice head was cleaned with 10% povidone-iodine after hair shaved. A 0.5-cm-long middle incision was made to expose skull and bregma that was set as coordinate zero (x=0, y=0, z=0). A 5 mm diameter craniotomy was made in predetermined coordinates (x=-0.34 mm, y=-1.0 mm, z=0) to place catheter that inserted into a depth of 2.5 mm. Then, the catheter was fixed by acrylic dental cement, and the incision was closed. Mice were rested for at least 7 days before another operation.

### Mice model of cecal ligation and puncture (CLP)

Mice were laid on the operating table after anesthetized with chloral hydrate (5%, 30 mg/kg of body weight). A 1-cm-long middle abdominal incision was made to expose the cecum which was ligated below the ileocecal valve, and then punctured once with 21-gauge needle. A bit of feces was extruded by compressing the ligated cecum slightly. The cecum was placed back into peritoneal cavity, followed with incision closed. All mice received fluid resuscitation by subcutaneously injecting 1 ml normal saline. While sham-operated mice had cecum isolated and then replaced it back into abdominal cavity without ligating and puncturing.

### Experimental design and intracerebroventricular (ICV) injection

Mice were randomly divided into four groups: sham CLP group (sham-1), CLP group, sham ICV injection group (sham-2), CLP plus ICV injection group (BoxA, 1 μg). The CLP groups and the CLP plus BoxA groups were both further divided into two groups according to the sacrificed time points of 24 and 48 hours after CLP surgery (Sepsis 24h+BoxA, Sepsis 48h+BoxA). In order to inhibit brain HMGB1 directly, the injection of BoxA solution (0.25 μg/μl, with 4 μl saline solution) was conducted at 0, 24 and 48 hours after operation. A pre-assembled syringe needle was placed into the cannula in accordance with its length. Then, the syringe was removed at 5 minutes after injection. Mice of sham group were injected with 4 μl normal saline (sham-2). The survival rates of all groups were observed for 7 days after CLP surgery.

### Immunofluorescence staining

Mice were sacrificed by giving an overdose of anesthetic at determined time points. Brain tissues were post-fixed in 4% paraformaldehyde solution, and dehydrated by ethanol gradient. The 5-μm coronal sections were made and boiled in citrate solutions for antigen retrieval. They were incubated with 0.3% Triton and blocked with goat serum for 2 hours. Then samples reacted with primary antibody at 4°C over night, followed by incubating with secondary antibody. The sections were imaged by inversion fluorescence microscope and analyzed by Image-Pro Plus 6.0 software.

### Western blotting

Brain tissues were homogenized in ice-cold RIPA lysis solution, and further centrifuged (4°C, 12000 rpm for 30 minutes) to collect supernatants which were quantified through BCA detection kit. 100 μg samples were separated on 10% SDS/polyacrylamide gel, and then transferred to polyvinylidene fluoride membranes. Membranes were blocked by 10% nonfat milk and incubated with primary antibody at 4°C over night, which further reacted with secondary antibody at room temperature for an hour. The membranes were measured and analyzed by Investigator ProImage system after incubated with ECL detecting kit.

### Hematoxylin-eosin staining

Brain tissues were embedded with paraffin after being dehydrated. These samples were cut into 5-μm coronal sections and paved on glass slides. Then, the sections were stained with hematoxylin-eosin (HE), and imaged by light microscope.

### S100β and neuron-specific enolase (NSE) determination

Levels of S100β and NSE in periphery blood were measured by enzyme-linked immunosorbent assay (ELISA). Serum samples were collected and quantified with ELISA kits in accordance with the manufacturer's instructions.

### TUNEL staining

The coronal slices (5 μm) of brain tissues were made after paraffin-embedded. Neuron apoptosis was measured by using TUNEL kits (Roche, USA) following the manufacturer's protocols, and analyzed by inversion fluorescence microscope.

### Behavior assessment

Behavior changes were evaluated by open field tests and Morris water maze (MWM) tests. Mice were arranged for behavior tests on the 7th day after CLP, and all tests were performed between 8:00 am and 17:00 pm.

#### Open field test

The white open field consisted of a 50 cm × 50 cm area open square with 30 cm high walls. Mouse was placed singly in the center of the field, and observed for 5 minutes to assess its locomotor activity. Testing videos were analyzed by Any-Maze software.

#### Morris water maze

The MWM for mouse was formed with 150 cm in diameter and 50 cm in depth, filled with water (23°C, 40 cm in depth). In training trials, mice were placed in water from different quadrants to find the hided platform within 60 seconds, and guided to the platform if they failed. Each mouse was arranged for 6 trials a day with 25 minutes inter-trial interval. Then, mouse was given a probe test with platform removed at 24 hours after 5 days training trials. It was released from the west quadrant, and allowed to swim within 60 seconds with swimming paths recorded.

### Statistical analysis

Data were analyzed by SPSS20.0, and presented as mean ± standard deviation (SD). Survival rates were analyzed by Kaplan-Meier survival curves. One-way analysis of variance (ANOVA) was used for assessing differences among multiple groups, student t-test was applied to make comparisons for intergroup, and log rank test was used for comparing survival. Significant statistical difference was considered when P values <0.05.

## Abbreviations

HMGB1: high mobility group box-1 protein
CLP: cecal ligation and puncture
HE: hematoxylin-eosin
TUNEL: TdT mediated dUTP nick end labeling
IL-1β: interleukin 1β
TNF-α: tumor necrosis factor-α
IL-6: interleukin 6
TNFR1: TNF receptor 1
ICV: intracerebroventricular
NSE: neuron-specific enolase
ELISA: enzyme-linked immunosorbent assay
MWM: Morris water maze
ANOVA: one-way analysis of variance
BBB: blood brain barrier
CSF: cerebrospinal fluid
